# An *in vitro* and *ex vivo* wound infection model to test topical and systemic treatment with antibiotics

**DOI:** 10.1111/jam.15756

**Published:** 2022-08-09

**Authors:** Yanyan Cheng, Paul A. De Bank, Albert Bolhuis

**Affiliations:** ^1^ Department of Pharmacy and Pharmacology and the Centre for Therapeutic Innovation University of Bath Bath UK

**Keywords:** antibiotics, electrospinning, *Staphylococcus aureus*, wound dressing, wound infection model

## Abstract

**Aims:**

This study aimed to develop a wound infection model that could be used to test antibiotic‐loaded electrospun matrices for the topical treatment of infected skin and compare the effectiveness of this treatment to systemically applied antibiotics.

**Methods and Results:**

3D‐printed flow chambers were made in which *Staphylococcus aureus* biofilms were grown either on a polycarbonate membrane or explanted porcine skin. The biofilms were then treated either topically, by placing antibiotic‐loaded electrospun matrices on top of the biofilms, or systemically by the addition of antibiotics in the growth medium that flowed underneath the membrane or skin. The medium that was used was either a rich medium or an artificial wound fluid. The results showed that microbial viability in the biofilms was reduced to a greater extent with the topical electrospun matrices when compared to systemic treatment.

**Conclusions:**

An *ex vivo* infection model was developed that is flexible and can be used to test both topical and systemic treatment of wound infections. It represents a significant improvement over previous *in vitro* models that we have used to test electrospun membranes.

**Significance and Impact of the Study:**

The availability of a relatively simple wound infection model in which different delivery methods and dosage regimes can be tested is beneficial for the development of improved treatments for wound infections.

## INTRODUCTION

Infected wounds, in particular those that fail to heal in a timely manner, are a significant burden for patients and affect their quality of life. In addition, the treatment of these wounds costs more than that of typical wounds and can lead to prolonged or excessive inflammation, the development of chronic infections and the failure of dermal and epidermal cells to react to reparative stimuli (Frykberg & Banks, 2015). Biofilms on the wound bed, which slow healing and increase resistance to antimicrobial treatment, are often polymicrobial and can include fungi, viruses and/or protozoa, as well as multi‐species bacterial communities (Dowd et al., 2008; Lipsky & Hoey, 2009; Roy et al., 2014). For bacterial infections, *Staphylococcus*, *Enterococcus*, *Pseudomonas*, *Proteus*, *Citrobacter*, *Streptococcus*, *Micrococcus*, *Escherichia*, *Peptoniphilus*, *Enterobacter*, *Stenotrophomonas*, *Finegoldia* and *Serratia* are the most commonly reported isolates in chronic wounds (Lipsky & Hoey, 2009; Rahim et al., 2017).

Biofilm models, both static and dynamic, have been designed to replicate wound bed micro‐environments *in vitro* and in vivo for the study of infection biology and testing the efficacy of novel wound therapies, including new drugs and formulations (Brackman & Coenye, 2016). The latter includes advanced drug delivery systems such as controlled release matrices, which can deliver topical antibiotics directly to the wound, overcoming possible side effects caused by large systemic doses and improving drug efficacy (Calamak et al., 2017; Miguel et al., 2019). In vivo biofilm models have also been developed in previous years, such as models using mice (Akiyama et al., 1996; Rumbaugh et al., 1999), rabbits (Gurjala et al., 2011) and pigs (Pechter et al., 2012; Roche et al., 2012). Rodent models are easier to set up, but the skin of rodents is very different from that of humans, and wound healing in rodents occurs via a different process (Klein et al., 2018). However, porcine skin resembles human skin in many aspects, for example, in its anatomy, physiology and biochemistry (Herkenne et al., 2006; Klein et al., 2018), making porcine skin a valuable in vivo model to mimic human infected wounds. However, in vivo experiments with pigs are expensive, time‐consuming and difficult to employ at scale, which may be necessary to test a wide range of treatment options.

In contrast to in vivo models, *in vitro* models are generally more scalable, less expensive and do not have an ethical burden (Yang et al., 2013). When considering wound infection models, an *in vitro* biofilm model for studying wound infection should consider growth on a semi‐solid surface with nutrients supplied at the bottom of the biofilm, oxygen from the top at an interface that is exposed to air and a flow rate that mimics the production of wound exudate (Agostinho et al., 2011; Brackman & Coenye, 2016). As a simple option, the colony biofilm model can be a first step towards building a more complex model. In this model, a biofilm develops on a membrane that can be easily transferred from one agar plate to another (Merritt et al., 2011). Other models have also been used such as the drip‐flow reactor, in which a biofilm is grown in a chamber with a continuous nutrient supply (Agostinho et al., 2011; Xu et al., 1998), or the Lubbock wound biofilm model, in which a wound exudate‐like medium was used with bacteria growing on plastic tips or silicone discs (Sun et al., 2008). More recently, a novel flow system using 3D‐printing technology was established by Duckworth et al. (2018) and was successfully used in the development of single‐ and double‐species *S. aureus* and *P. aeruginosa* biofilms and treatment with antimicrobial dressings.

Additionally, *ex vivo* models have been employed using explanted skin. Whilst explanted human skin is an excellent model (Yoon et al., 2019), it suffers from potential problems with sourcing or costs. A good alternative is porcine skin, as its structure is very similar to that of human skin (Herkenne et al., 2006), it can be obtained from slaughterhouse material so is ethically neutral and adheres to 3Rs principles (replace, reduce, refine) and is well established in research on topical delivery of drugs. We previously reported the use of this tissue to grow *S. aureus* biofilms, which were then treated with controlled‐release electrospun matrices containing the antibiotic tetracycline (Alhusein et al., 2016). However, this model was rather basic in that the explanted pig skin was incubated on nutrient‐rich agar plates. In this research, we aimed to further explore this approach to develop a model which more accurately mimics the conditions in a skin wound by, firstly, using an artificial wound fluid (AWF) that contains nutrients more similar to those found in a wound environment (Frohm et al., 1996; Trengove et al., 1996). Secondly, we used a 3D‐printed device that enables fluid flow, to simulate the dynamic environment of a wound. Specifically, the focus was on developing a flow system using 3D‐printed flow chambers that could be used to examine the killing effects of electrospun matrices loaded with antibiotics that are commonly used for topical treatment of infected skin.

## MATERIALS AND METHODS

### Chemicals

All chemicals were purchased from Sigma‐Aldrich or Fisher Scientific, unless otherwise specified.

### Cultures and growth conditions

The bacterial strains used were *Staphylococcus aureus* NCTC 6571 and methicillin‐resistant strain *S. aureus* MRSA252 (Holden et al., 2004), which were maintained on tryptic soy agar (TSA; Oxoid, Basingstoke, UK). Overnight cultures were grown from single colonies in tryptic soy broth (TSB; Oxoid) at 37°C with shaking at 200 rpm.

### Artificial wound fluid (AWF)

The composition of AWF was as follows: 5% heat‐inactivated horse serum, 0.36% NaCl, 0.1% sodium lactate, 0.1% glucose, 0.054% KH_2_PO_4_, 0.05% NaCO_3_, 0.03% casamino acids, 0.02% sodium citrate, 0.02% MgCl_2_·6H_2_O, 0.02% thiamine·HCl, 0.02% nicotinic acid, 0.02% FeSO_4_·7H_2_O, 0.01% CaCl_2_·2H_2_O and 0.01% urea. Where indicated, 50 mg/L ampicillin and 20 mg/L kanamycin were added to cultures of *S. aureus* MRSA252 in TSB or AWF, to avoid contamination from the skin pieces.

### Minimal inhibition concentrations of antibiotics

Minimum inhibitory concentration (MIC) values of vancomycin hydrochloride, tetracycline hydrochloride, gentamicin and fusidic acid against planktonic *S. aureus* MRSA252 and NCTC 6571 were determined with a microdilution broth media method (Andrews, 2001) using Mueller Hinton broth (MHB), TSB or AWF.

### Sterilization and histological staining of porcine skin

Porcine skin was obtained from slaughterhouse material, as outlined previously (Ho et al., 2020). The use of the skin was approved by the Animal Welfare Ethical Review Body (AWERB) of the University of Bath. Dermatomed skin pieces, of approximately 750 μm thickness, were cut into approximately 1 × 1 cm square size for further use. Skin pieces were then cleaned with Milli Q water and hairs were removed by carefully cutting with small scissors. The skin was then sterilized with peracetic acid (PAA), following a procedure similar to the one described previously (Huang et al., 2004). Briefly, porcine skin pieces were immersed in 0.5% PAA in phosphate‐buffered saline (PBS) with a final pH of 7.0, for 3 h with gentle shaking at room temperature. Next, the skin pieces were washed three times for 15 min each with PBS. All sterilized skin pieces were then placed on TSA plates and incubated at 37°C for 3 days to evaluate the efficacy of sterilization. Finally, histological staining was performed using haematoxylin and eosin stain (H&E stain) as detailed in (Alves et al., 2018) to evaluate the damage to skin structures after sterilization. The PAA‐treated skin was compared to skin that was untreated or washed in only PBS instead of PAA.

### Biofilms on porcine skin

An open, round wound was made on the epidermal side of porcine skin with a 2 mm biopsy punch before sterilization. Following sterilization of the skin pieces, 1 μl aliquots of 1:100 diluted overnight culture of *S. aureus* MRSA252 was inoculated on the wound bed and then dried for 15 min before transferring onto TSA or AWF agar (AWFA) plates containing 15 μg/ml ampicillin and 20 μg/ml kanamycin sulphate. The small volume of diluted *S. aureus* culture was necessary here to prevent flow‐off from the porcine skin, and the combination of ampicillin and kanamycin, as used previously (Alhusein et al., 2016), was adequate to suppress the growth of any contaminants on the skin that were not killed by the PAA treatment. The inoculated skin pieces were incubated at 37°C for 2 days and then treated with electrospun matrices loaded with antibiotics (see below). After treatment, each porcine skin piece was gently washed twice with 10 ml sterile PBS to remove planktonic cells and then transferred into a Bijou bottle containing 2 ml PBS. The bottles were sonicated for 15 min, followed by vortexing for approximately 60 s to mechanically detach the bacterial cells from the skin. Finally, serial dilution was performed for a viable count of *S. aureus* MRSA252.

### Poly‐ε‐caprolactone (PCL) electrospun nanofibrous matrices loaded with antibiotics

PCL/antibiotic mixtures were prepared by dissolving PCL (Mn 70,000–90,000 by GPC) in a solvent mixture, followed by the addition of antibiotics (Table 1). The suspension was stirred overnight until required for electrospinning. The polymer suspension was loaded into a gas‐tight syringe (1005TLL, 5.0 ml SYR; Hamilton, Bellefonte, USA) and electrospun in a vertical configuration at 17–19 kV and a flow rate of 0.6–1.0 ml/h, controlled by a syringe pump (Cole Parmer, 116805), with a distance between the tip of the needle and the grounded, aluminium foil‐covered collector of 18 cm. After electrospinning, the randomly oriented nanofibrous matrices were placed in a safety cabinet overnight, and then carefully peeled from the foil and cut into 0.5 × 0.5 cm square pieces with a scalpel. The matrices were stored in Petri dishes at room temperature for later use.

**TABLE 1:** Parameters of preparing antibiotics‐loaded electrospun nanofibrous matrices

Electrospun nanofibrous matrices	Antibiotics	Solvent	Concentration of polymer in solvent (w/v)	% of antibiotics (w/w of polymer)	High voltage (kV)	Flow rate (ml/h)
PCL	Gentamicin	3:1 v/v chloroform: methanol	11%	3%	19	0.6
PCL	Tetracycline	3:1 v/v chloroform: methanol	12%	5%	17	0.8
PCL	Fusidic acid	3:1 v/v chloroform: methanol	14%	5%	19	1.0
PCL/SF	Gentamicin	SF: 1,1,1,3,3,3‐hexafluoro‐2‐ propanol (HFIP); PCL: 3:1 v/v dichloromethane: HFIP	10% SF with 10% PCL mixed in a ratio of 50:50 (v/v)	3%	19	2.0

Silk fibroin (SF) was prepared from *Bombyx mori* silk cocoons following a previously published procedure (Luetchford et al., 2020). Then, polymers were prepared by dissolving SF (10% w/v) in 1,1,1,3,3,3‐hexafluoro‐2‐propanol (HFIP) and PCL (10% w/v) in a 3:1 v/v mixture of dichloromethane: HFIP. Both solutions were left under stirring overnight before mixing in a ratio of 1:1 (v/v). Then, the suspension was left under stirring for 24 h before adding 3% gentamicin sulphate (GS; w/w of the total polymer weight). Finally, the PCL/SF/GS solution was left under constant stirring for at least 3 h prior to electrospinning (Table 1).

### Disc susceptibility test

Electrospun membranes were punched into 6 mm discs and placed on TSA plates that were seeded with ~10^5^ colony‐forming units (CFU) of *S. aureus* MRSA252. The agar plates were incubated at 37°C, and after 24 h, the zone of inhibition (ZOI) around the discs was measured.

### Design, set up and application of flow system

**FIGURE 1:**
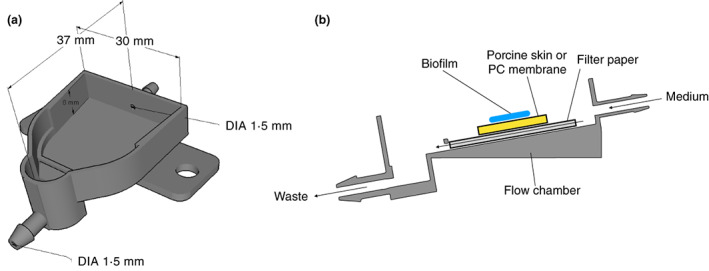
Schematic image of 3D‐printed flow chamber with a 10° slope. Dimensions of the flow chamber are shown in a, and a side view of the setup showing the flow of medium through the chamber (arrows) is shown in b.

Flow chambers, with a 10° slope (Figure 1), were designed using SketchUp Make (Trimble) and were printed from polylactic acid (2.85 mm, Ultimaker™) at 200°C using an Ultimaker™ 2+ 3D printer with 0.15 mm layer height, 30% infill and a speed of 60 mm/s. The final design of the flow chamber was based on earlier models that were improved iteratively, through a trial‐and‐error process, by testing, for instance, flow of growth media through the device and capability of holding different substrates for growing biofilms in place. After printing, chambers were sterilized by immersion in 0.1% PAA with shaking at room temperature for 1 h, followed by washing with PBS 3 times for 15 min each. Then, a layer of sterilized filter paper (~3 × 3cm) was placed into each flow chamber, and a 13 mm polycarbonate (PC) membrane or porcine skin piece seeded with *S. aureus* MRSA252 was placed on the top of the filter paper. The chambers were connected to a multi‐channel peristaltic pump (530 series pump with 12‐channel pump head; Watson‐Marlow Pumps) and growth media, TSB or AWF, were supplied at 0.04 ml/min, enabling bacterial growth in 12 flow chambers simultaneously. Biofilms in the flow chambers were grown for 48 h and subsequently treated for 24 h by placing antibiotic‐loaded electrospun nanofibrous matrices on top of them. A viable count was then performed to determine the killing effect of released antibiotics. To mimic systemic treatment, antibiotics were added to the growth medium, followed by the determination of the viable count in the biofilms.

### Confocal laser scanning microscopy (CLSM) imaging of *S. aureus* MRSA252 biofilms

PC membranes seeded with *S. aureus* MRSA252 were placed in the flow system as above, and samples were taken from time points of 1, 4 and 8 h. Membranes with biofilms were washed gently with Ringer's solution to remove planktonic cells, followed by staining with LIVE/DEAD™ *Bac*Light™ Bacterial Viability Kit (Fisher Scientific) according to the manufacturer's instructions. After staining, samples were gently washed twice with Ringer's solution to remove excess stain and then transferred into confocal dishes with a 15 mm glass bottom (non‐treated; VWR) for imaging with CLSM (LSM880, ZEISS).

### Statistical analysis

Most experiments in this research were performed in triplicate with two technical repeats. The mean values of technical repeats were used for the statistical analysis. Data entry and calculations were performed using Microsoft Excel, and statistical analysis was carried out using GraphPad Prism 9 (GraphPad Software Inc.) using a *t*‐test or a one‐way analysis of variance (ANOVA).

## RESULTS

### Comparison of antibiotic resistance in AWF with that in rich culture media

One requirement for this wound model was to use a nutritional environment that was more similar to that of a real wound in comparison to standard laboratory media. For that reason, we used an artificial wound fluid (AWF) which was similar to other published recipes see, for example, (Ohlknecht et al., 2017), with the inclusion of whole serum instead of albumin. In addition, because of the nutritional requirements of the *S. aureus* strains used, additional components such as vitamins and amino acids were added. Growth of *S. aureus* was slower in AWF compared to TSB and did not support growth to similar cell densities (Figure S1a), but was nevertheless at an acceptable level for our experiments.

The sensitivity of *S. aureus* against antibiotics commonly used for topical wound treatment (tetracycline, gentamicin and fusidic acid) was tested in AWF and compared to that in TSB, a standard culture medium for *S. aureus*, and MHB, which is routinely used for testing antibiotic sensitivity. Two strains were tested: *S. aureus* MRSA252, a clinical isolate, and the reference strain *S. aureus* NCTC 6571. The two strains had similar MIC values when compared to each other but, depending on the antibiotic, there were significant differences when comparing the different media (Table 2). The *S. aureus* strains were more sensitive to gentamicin in AWF in comparison to the other media, whereas the opposite was observed for tetracycline and fusidic acid.

**TABLE 2:** Determination of MICs by microdilution of gentamicin, tetracycline and fusidic acid (mg/L) in MHB, TSB and AWF

Medium	Gentamicin		Tetracycline		Fusidic acid	
	MRSA252	NCTC6571	MRSA252	NCTC6571	MRSA252	NCTC6571
MHB	4	4	0.25	0.5	0.25	0.5
TSB	16	32	0.5	0.5	0.25	2
AWF	0.125	0.125	8	8	16	16

### Bacterial growth on sterilized porcine skin

Another component of our model was the incorporation of explanted porcine skin, which, to avoid the growth of other microbes in our experiments, was sterilized using 0.5% PAA. To ensure that the sterilization process did not damage the structure of the skin, histological staining was performed, which showed no obvious visual differences between skin treated with PAA and skin that was left untreated or treated with PBS (Figure S2). One problem was that treatment with PAA did not always prevent unwanted microbial contamination, but this could be overcome by the addition of 50 mg/L ampicillin and 20 mg/L kanamycin to the growth media. The *S. aureus* MRSA252 strain is resistant to these antibiotics, which do not affect its rate of growth in either TSB or AWF, apart from a slightly longer lag phase in the presence of ampicillin (Figure S1b).

**FIGURE 2:**
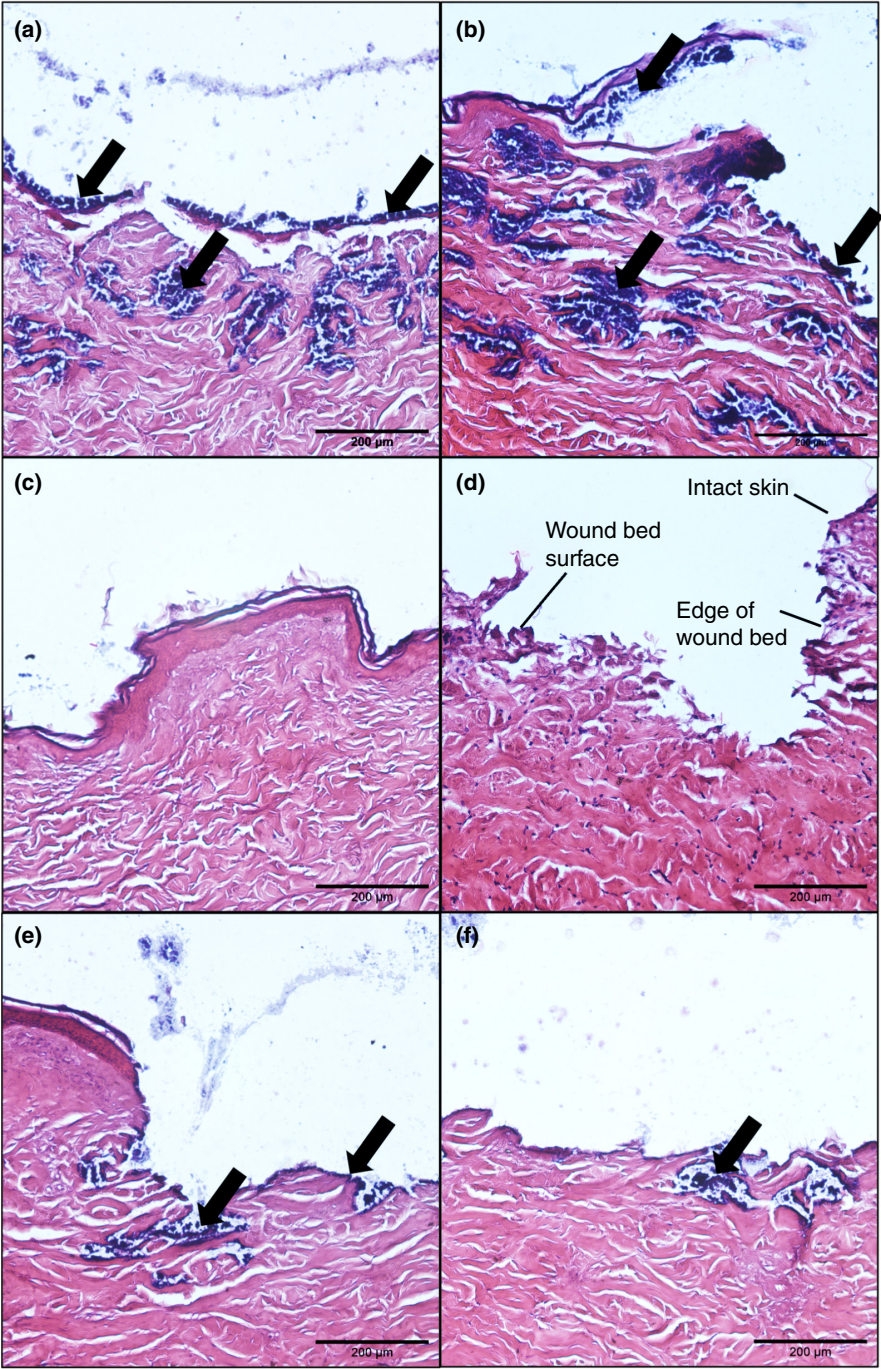
Histological images of punctured porcine skin placed on TSA (a, b, c and d), or AWFA (e and f) with/without *Staphylococcus aureus* MRSA252. Panels a, b, e, and f show infected wound beds; panel c shows infected skin but outside the area where the biopsy was made, and panel d shows uninfected skin. Black arrows show areas of skin with growth of *S. aureus* MRSA252. The scale bar is equivalent to 200 μm.

**FIGURE 3:**
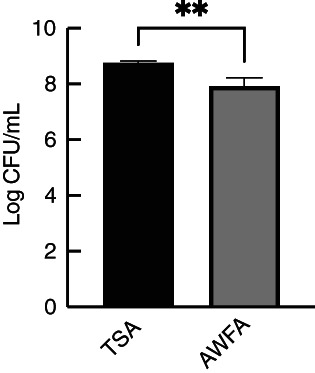
Viable counts of *Staphylococcus aureus* MRSA252 grown on porcine skin pieces, incubated on TSA or AWFA. Experiments were performed in triplicate with two technical repeats, and the error bars shown are standard deviations. An unpaired *t‐*test was used to compare the mean of the two groups (**p < 0.01).

To simulate a wound, porcine skin pieces were damaged with a biopsy punch and seeded with *S. aureus* MRSA252. The skin pieces were then placed on TSA or AWFA for 5 days, after which the infected skin was analysed by histological staining and determination of the viable count. When the skin pieces were incubated on TSA, bacterial growth was visible on damaged skin (Figure 2a,b), whereas no growth was seen outside the wound area (Figure 2c). For comparison, uninfected skin is shown in Figure 2d. When the skin pieces were placed on AWFA, bacterial growth was also observed on the wound bed (Figure 2e,f), although this appeared to be reduced in comparison to skin incubated on TSA. This was confirmed by the recovery of *S. aureus* from the porcine skin and determination of the viable count; there was a significant difference between the viable count of biofilms grown on porcine skin placed on TSA compared to AWFA, with the CFU/mL being six‐fold lower in the latter (Figure 3).

### Development of *S. aureus* MRSA252 biofilms in a 3D‐printed flow system

**FIGURE 4:**
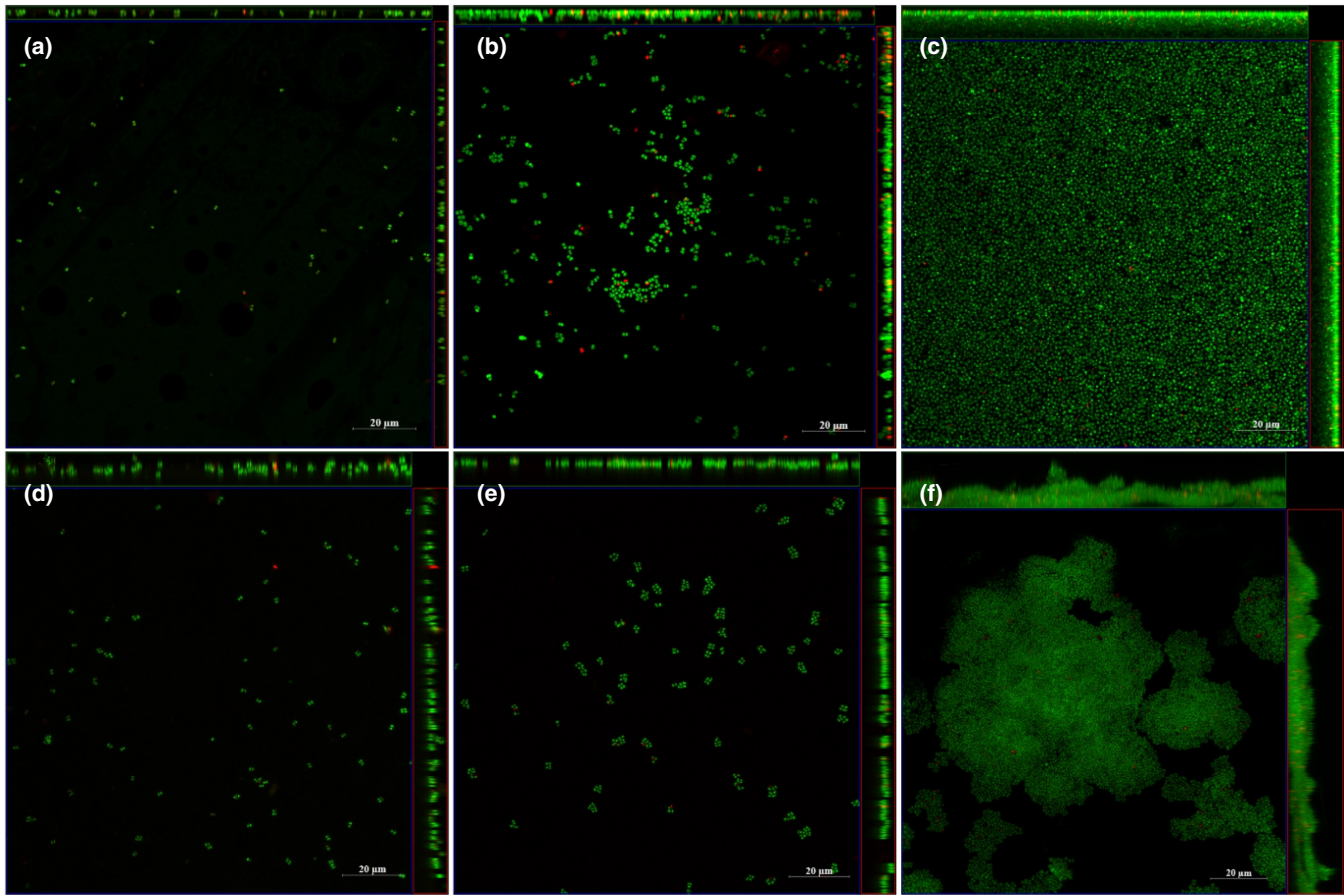
Representative images of ortho‐ and side‐view images of *Staphylococcus aureus* MRSA252 biofilm grown on PC membranes in the flow system with TSB (a, b, and c) and AWF (d, e and f), where green indicates live cells and red indicates dead cells. The images were captured at 1 h (a, d), 4 h (b, e), and 8 h (c, f) of biofilm growth in the flow system. The scale bar is equivalent to 20 μm.

An important aim of this study was to develop a 3D‐printed flow chamber that could be used to grow biofilms on the skin and test the effects of both topically and systemically applied antibiotics on an infected skin wound. The flow chambers were printed from PLA and had an internal volume of approximately 3 ml, with a slope of 10° to allow growth media to flow out under gravity. The flow reflects the dynamic state of a wound, with the rate chosen (2.4 ml/h) in a range similar to other dynamic flow models (Brackman & Coenye, 2016). A pump with a 12‐channel pump head was used to enable the simultaneous attachment of 12 flow chambers. PC membranes were inoculated with *S. aureus* and then placed on sterile filter paper in the flow chambers. Growth medium (TSB or AWF) was then slowly pumped into the chamber in such a manner that it flowed through the filter paper, underneath the biofilm substrate, thereby mimicking fluid flow in underlying tissue. The formation of *S. aureus* MRSA252 biofilms on the PC membranes was then visualized at different time points using LIVE/DEAD *Bac*Light staining and confocal microscopy. After a 1‐h incubation, some adherent *S. aureus* MRSA252 cells could be seen on the membrane when using either TSB or AWF as the growth medium (Figure 4a,d). After 4 h of incubation with TSB, biofilm formation was at an early stage with some microcolonies being formed, whilst with AWF, there were fewer and smaller microcolonies (Figure 4b,e). Finally, after 8 h, a uniform biofilm covered the entire surface when grown on TSB (Figure 4c), whilst with AWF, a multi‐layered structure was formed that was more irregular and did not cover the entire membrane (Figure 4f).

### Mimicking topical and systemic antibiotic treatments in the flow system

The effect of topical antibiotic treatment on *S. aureus* MRSA252 biofilms grown on PC membranes or explanted porcine skin in flow chambers was tested with electrospun antibiotic discs generated in‐house. Conditions for electrospinning were optimized for each antibiotic, to obtain matrices that showed continuous fibres with no beading, similar to our previous studies (Alhusein et al., 2016; Alhusein, Blagbrough, & De Bank, 2013). The antibiotic loading in the nanofibres was then determined by LC/MS. Because the electrospinning parameters were not identical for each antibiotic, 6 mm discs resulted in different amounts per disc (Table 3). The discs were also tested by placing these on agar plates inoculated with *S. aureus* MRSA252 and then measuring the ZOI the next day (Table 3). Fusidic acid showed the largest ZOI, whilst that of gentamicin was the smallest. The discs were also tested for sustained release of antibiotics by using the discs that were tested for their ZOI on a second plate that was freshly inoculated with *S. aureus* MRSA252. However, no ZOI was observed on the 2nd day, indicating that all antibiotics were released from the electrospun membranes on day 1.

**TABLE 3:** Loading and activity of electrospun antibiotic discs

Antibiotic	Drug per disc (μg)	ZOI (mm)
Gentamicin	3.3 (±0.2)	15.7 (±1.8)
Tetracycline	7.4 (±0.6)	30.0 (±1.9)
Fusidic acid	12.3 (±2.0)	31.8 (±1.3)

**FIGURE 5:**
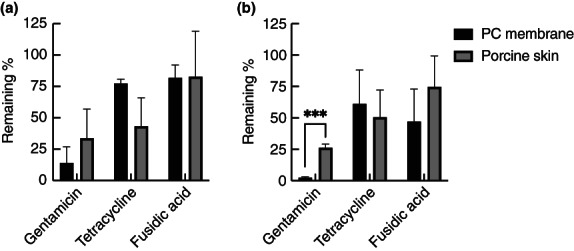
Effect of topically applied antibiotics on *Staphylococcus aureus* MRSA252 biofilms in the flow system. Biofilms were grown for 48 h in TSB (a) or AWF (b), and then electrospun matrices containing antibiotics were placed on top for 24 h. The number of viable cells is expressed as the % of viable cells compared to controls without antibiotics. The experiments were performed in triplicate, with two technical repeats in each, and the error bars shown represent standard deviations. Black bars: biofilms grown on PC membranes; grey bars: biofilms grown on porcine skin. Statistical analysis was performed using unpaired *t*‐tests with a False Discovery Rate correction (***p < 0.001).

These discs were placed for 24 h on top of 2‐day‐old biofilms grown in the flow chambers, after which the discs were carefully removed and the number of viable bacteria on the membranes or skin determined. When biofilms were grown on PC membranes, gentamicin was more effective against the biofilms compared to tetracycline or fusidic acid in both TSB and AWF (Figure 5), even though the gentamicin discs contained the lowest quantity of antibiotic and gave the smallest ZOI on agar plates. In skin, this same trend was also observed but was less obvious because gentamicin was less effective in treating skin‐grown biofilms. For instance, with AWF, there was a 10‐fold increase in viable bacteria remaining on the skin (23.6%) in comparison to PC membranes (2.6%). It should be noted, however, that the combination of AWF and treatment with gentamicin led to small colony variants (SCVs), which showed up only after prolonged incubation of the TSA plates that were used for determining the viable count. Such SCVs could lead to an underestimation of the number of viable bacteria.

**FIGURE 6:**
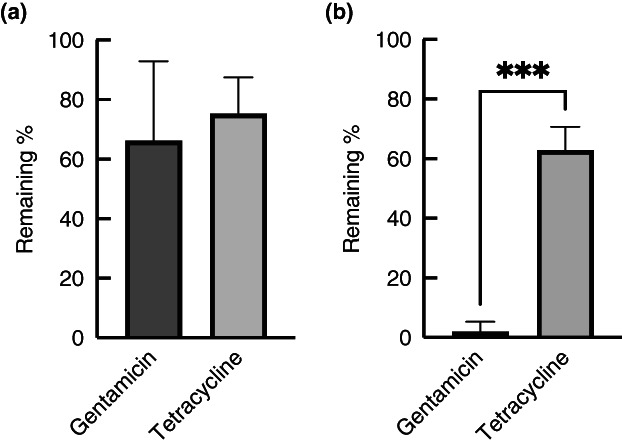
Effect of mimicking systemic delivery of antibiotics to *Staphylococcus aureus* MRSA252 biofilms in the flow system. Biofilms were grown on PC membranes for 48 h in either TSB (a) or AWF (b) in the flow device, and then antibiotics were added to the growth medium that flows underneath the biofilm for 24 h. The number of viable cells is expressed as the % of viable cells compared to controls without antibiotics. The experiments were performed in triplicate, with two technical repeats in each, and the error bars shown represent standard deviations. Statistical analysis was performed using an unpaired *t*‐test (***p < 0.001).

To mimic systemic antibiotic treatment of biofilm grown in the flow system, 5 mg/L gentamicin or 1.6 mg/L tetracycline was added into growth media after 2 days of biofilm formation on PC membranes; the concentrations used are similar to the peak concentrations that are reached in serum with systemic treatment (Agwuh & MacGowan, 2006; Barza & Lauermann, 1978). This was not tested with fusidic acid, as this is mainly used for topical treatment. In TSB, *S. aureus* MRSA252 biofilms were tolerant to both antibiotics, with 66 and 75% of viable cells remaining viable after 24 h of treatment with gentamicin and tetracycline, respectively (Figure 6). In AWF, biofilms were also tolerant to tetracycline (63% viable cells remaining), but gentamicin was efficient in killing cells in biofilms, with only 2% of cells surviving the treatment.

The electrospun PCL membranes loaded with antibiotics failed to show sustained release of these antibiotics (see above). Therefore, a different nanofibre membrane that was generated from a blend of PCL and silk fibroin (SF) was tested. This was performed with gentamicin only, as this was the antibiotic that showed the best activity in the experiments above. The gentamicin loading efficiency could not be tested with the PCL/SF membranes, as the matrices did not fully dissolve after electrospinning the membranes. The ZOI obtained from these was slightly higher than that obtained with PCL only (17.8 mm for the PCL/SF membrane and 15.7 mm for the PCL membrane), indicating that the loading efficiency was similar or slightly higher. However, these membranes also exhibited a lack of sustained antibiotic release, as there was no ZOI when the membranes used on one plate were transferred to a second plate.

**FIGURE 7:**
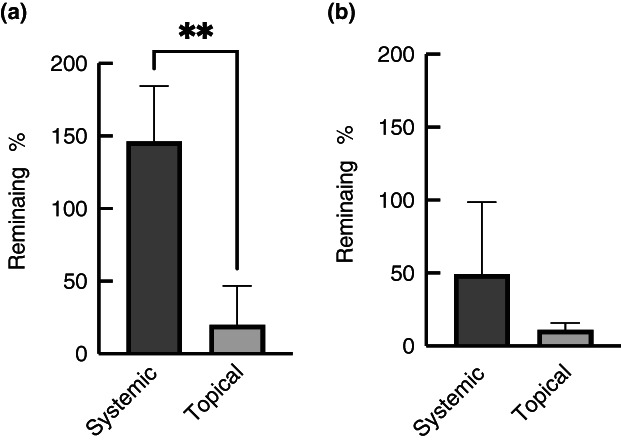
Effect of systemic and topical treatment of infected porcine skin. The skin was inoculated with *Staphylococcus aureus* MRSA252 and grown for 2 days in the flow cells using either TSB (a) or AWF (b). The biofilms were then treated topically with gentamicin (drug‐loaded electrospun PCL/SF matrices) or systemically (by adding gentamicin to the growth medium flowing underneath the skin) for 24 h. The experiments were performed in triplicate, with two technical repeats, and error bars are standard deviations. Statistical analysis was performed using an unpaired *t*‐test (**p < 0.01).

The gentamicin‐loaded PCL/SF membranes were used to compare the effects of systemic and topical treatment in the flow system using infected porcine skin. When the biofilms were grown on TSB, topical treatment resulted in a significant decrease in the number of viable bacteria that could be recovered (20% of untreated control), whilst systemic treatment resulted in a further increase in the number of bacteria on the skin (146% of the control). The difference between topical and systemic treatment was statistically significant (*p* < 0.01). When the biofilms were grown in AWF (Figure 7b), topical treatment also appeared to be more effective (11% remaining as compared to 49% for systemic), but this was not statistically significant (*p* > 0.05). This indicates that in the setup used, gentamicin penetrates poorly into the porcine skin.

## DISCUSSION

In this study, a 3D‐printed flow chamber was designed to model infected wounds. This was developed to test the efficacy of electrospun matrices loaded with antibiotics, but the system can be used more generally to test both topical and systemic treatment of microbial biofilms that are grown either on a membrane, explanted skin or on other substrates. In developing this, we had a number of requirements, the first of which was to use a growth medium that mimicked the wound environment more closely than a standard nutrient‐rich medium. One issue is that AWF is not standardized (Brackman & Coenye, 2016), and many different recipes can be found in the literature. Our starting point was a previously published recipe (Ohlknecht et al., 2017), but we replaced albumin with whole serum. However, the nutritional requirements of the *S. aureus* strains used in this study necessitated the further addition of vitamins and amino acids, and the concentration of serum had to be limited as otherwise growth was extremely slow (data not shown). Growth in the AWF used here was still limited in comparison to TSB, but that was acceptable and perhaps even desirable as bacterial growth is also expected to be relatively slow in vivo compared to *in vitro* (White, 2001).

With the use of AWF, it was noted that, compared to standard laboratory media, tetracycline and fusidic acid had higher MIC values against *S. aureus*. This lower susceptibility is most likely explained by the slower growth of *S. aureus* in AFW as compared to TSB or MHB, a well‐known phenomenon that is illustrated by the increased tolerance to β‐lactam antibiotics in slow or non‐growing cells (Cozens et al., 1986; Tuomanen & Tomasz, 1990). In contrast, *S. aureus* was more sensitive to gentamicin in AWF in comparison to TSB or MHB (128 and 32‐fold less, respectively). Unlike tetracycline and fusidic acid, which enter cells through passive diffusion (Argast & Beck, 1984; Hancock & Bell, 1988), the uptake of gentamicin in *S. aureus* depends on the proton motive force (PMF) and the composition of the medium. For instance, one study found that *S. aureus* is more resistant to aminoglycosides in nutritionally richer media, which was related to a reduced uptake (Henry‐Stanley et al., 2014). Several factors may play a role in this, one of which is the ionic strength, which is known to correlate to tolerance to aminoglycosides (Beggs & Andrews, 1976). In addition, in nutrient‐rich media that support rapid growth and high cell densities, rapid oxygen depletion and acidification of the medium are expected, and both of these factors influence sensitivity to aminoglycosides (Schlessinger, 1988). This is possibly because anaerobiosis and a low pH reduce the Δψ component of the PMF, which is essential for gentamicin uptake (Mates et al., 1982). Taken together, it seems likely that the uptake of gentamicin is reduced in cells that are growing in TSB or MHB when compared to AWF, resulting in higher MIC values in TSB/MHB.

To grow biofilms in this study, we used either PC membranes, used commonly to grow a basic colony biofilm model or porcine skin pieces that were placed in 3D‐printed flow chambers. Porcine skin is a good mimic for human skin, because of its similarity in thickness, hair follicle density, pigmentation and collagen and lipid composition (Summerfield et al., 2015). The explanted porcine skin had to be sterilized, as otherwise natural microbes found on the skin of pigs would overgrow *S. aureus*. The preferred method was one that did little or no damage to the overall skin structure and, after a trial and error process, sterilization with PAA was chosen. Occasionally, microbial growth other than *S. aureus* was still observed on the porcine skin, and therefore, additional antibiotics to which the *S. aureus* MRSA 252 strain was resistant were added to the growth media.

If the skin is intact and healthy, *S. aureus* does not commonly cause infections, but if the local barrier is damaged, it can lead to a skin infection that could eventually result in more serious disease if it invades into deeper tissues (Bowler et al., 2001). In the porcine skin model, we did indeed observe that skin that was damaged with a biopsy punch was more easily infected with *S. aureus* MRSA 252 as compared to intact skin. This is most likely because, in damaged skin, extracellular matrix proteins become exposed, allowing *S. aureus* to interact through, for instance, collagen‐ and fibronectin‐binding proteins (Krishna & Miller, 2012). It should be noted that in the wound model used here, the infection is not controlled by an immune system, as we used dermatomed porcine skin that was stored frozen before use (Ho et al., 2020).

The 3D‐printed flow chambers that were used here have some similarities to other low‐shear flow systems that have been developed, such as the drip flow reactor (Goeres et al., 2009) and the Duckworth biofilm device (Duckworth et al., 2018). However, there are also some differences. Unlike the drip flow reactor where liquid flows on top of a biofilm that grows on a glass slide or other type of material, in the flow chambers used here the liquid flows through filter paper that is underneath the biofilm. Thus, nutrients come from below the biofilm, which is more similar to what would happen in an infected wound. The Duckworth device also has a similar arrangement with nutrient flow below the biofilm, but in that case, the biofilm is separated from the nutrients by an agar plug. The flow chambers designed by us have the advantage that they can accommodate skin pieces or other substrates that do not need to be regular‐sized, as long as they fit within the dimensions of the flow chamber. Furthermore, if combined with a multichannel pump, several flow chambers can be run at the same time which, if desired, each could have different conditions such as growth media or antimicrobial agents.

Having the different components of the infection model in place, electrospun nanofibrous matrices that were loaded with antibiotics were then evaluated using the 3D‐printed flow chambers. These membranes are similar to those that we had produced before (Alhusein et al., 2016; Alhusein, Blagbrough, & De Bank, 2013; Alhusein, De Bank, et al., 2013). In recent years, electrospinning techniques have been widely used in wound dressing development, and in this study, we developed membranes that were loaded with antibiotics used for topical treatment. These were applied to mature *S. aureus* MRSA252 biofilms grown in the flow chambers. The antibiofilm activity of the three antibiotics used did not follow their MIC values. For instance, both tetracycline and fusidic acid had considerably lower MIC values in TSB in comparison with AWF, but that difference was not seen when these antibiotics were used on biofilms. That lack of correlation between MIC values and antibiofilm activity is not particularly surprising and has been observed in several other studies (Macia et al., 2014). Furthermore, both tetracycline and fusidic acid were not particularly active on biofilms, with 50% or more of cells remaining viable after treatment, irrespective of whether the biofilms were grown on PC membranes or porcine skin. Better antibiofilm activity was seen with gentamicin, which appeared more effective in killing cells in *S. aureus* MRSA252 biofilms than tetracycline or fusidic acid. In addition, gentamicin killed more cells in biofilms grown on PC membranes compared to biofilms grown on the skin, in particular when using AWF as the growth medium. This is likely to be a reflection of the poor dermal penetration of gentamicin when applied topically (Oesterreicher et al., 2018). Unfortunately, none of the electrospun membranes that we created showed sustained release of antibiotics, as demonstrated by the lack of a zone of inhibition on agar plates when the membranes were, after use for 24 h, transferred to a second freshly inoculated plate. There are, however, strategies that could improve the release profile, such as generating multi‐layered membranes as shown in our previous studies (Alhusein et al., 2016; Alhusein, Blagbrough, & De Bank, 2013; Alhusein, De Bank, et al., 2013) or changing the polymers employed.

The flow chambers were also evaluated for testing systemic‐like treatment of biofilms, by including antibiotics in the growth medium that flows underneath the biofilms. Here, again gentamicin had good activity when using AWF as the growth medium, with only 2% of viable cells remaining. A direct comparison of systemic and topical treatment of infected porcine skin was performed only with gentamicin‐loaded PCL/SF matrices, which indicated that topical treatment was more effective than systemic treatment in the flow chambers. This is probably because systemic delivery into the skin also has its limitations; concentrations in subcutaneous adipose tissue are ~39% of peak serum concentrations (Lorentzen et al., 1996), and it could be expected that concentrations in the dermis or epidermis are lower than that. In a mouse model, others have also found that topical treatment was significantly better in eradicating *S. aureus* from wounded skin (Vingsbo Lundberg & Frimodt‐Moller, 2013). Considering that gentamicin has rather poor tissue penetration when delivered topically, the use of high doses is unlikely to lead to systemic side effects. This has indeed been shown previously, in a study where ultrahigh concentrations (>1000‐fold the MIC value) of gentamicin applied topically resulted in rapid decontamination of full‐thickness wounds whilst maintaining safe systemic levels (Junker et al., 2015). High concentrations of topically applied gentamicin may thus be an attractive option in the treatment of infected wounds.

It should be noted that gentamicin‐treatment of *S. aureus* MRSA252 biofilms (either in the topical or systemic‐like setup) resulted in SCVs when AWF was used as the growth medium, but not TSB. SCVs are a slow‐growing subpopulation of bacteria that are selected for by gentamicin (Edwards, 2012), as they are more resistant because of a deficiency in gentamicin uptake (Miller et al., 1980). They arise as a consequence of environmental stresses and are frequently observed in chronic recurrent infections (Kahl et al., 2016; Onyango et al., 2013). The reason that we observed SCVs when biofilms were grown in AWF but not in TSB is not clear. SCVs are present in any population of *S. aureus* cultures (Edwards, 2012) and it could be that, because the MIC of gentamicin is much lower with AFW‐grown cells as compared to TSB‐grown cells, a greater proportion of biofilm cells with the normal colony phenotype were killed in AWF, with the effect that SCVs are selected for these conditions. However, another explanation could be that the environmental stress of the AWF resulted in increased numbers of SCV. If that is correct, it would suggest that the AWF does indeed mimic clinical conditions where host‐induced stress can induce SCVs (Bui et al., 2015). However, it was beyond the scope of this study to investigate the appearance of SCVs in more detail.

In conclusion, we have successfully developed an *ex vivo* flow system to mimic *S. aureus* biofilm‐associated infected wounds. The model is a significant improvement on models we used previously to test electrospun matrices, as it is flexible and allows growing biofilms on multiple substrates including porcine skin. If conditions that mimic in vivo skin infections are desirable to grow biofilms, AWF is more suitable than general nutrient‐rich laboratory media as, based on the formation of SCVs, this medium appears to provide similar environmental stresses that are present in vivo. Importantly, our flow system can be used more widely in testing both systemic and topical delivery methods and dosage regimes of antibiotics or other antiseptics. Furthermore, due to the application of the 3D printing technique, our 3D print chambers can be modified to adapt to different experimental conditions. For instance, the printing materials could be changed and the size of the chamber could be modified. Several further improvements to the system could also be made, such as growing multi‐species biofilms or growth of the biofilms over a longer period of time to more accurately mimic chronic wound infections.

## CONFLICT OF INTEREST

The authors have no conflicts of interest to declare.

## Supporting information


Figure S1
Figure S2
