# Supplementary material for: An in vitro and ex vivo wound infection model to test topical and systemic treatment with antibiotics
Source: J Appl Microbiol. 2022 Aug 9;133(5):2993–3006. doi: 10.1111/jam.15756 (PMC9804477; doi:10.1111/jam.15756)
Supplement: Supplementary file 1 — Figure S1 Figure S2 [file JAM-133-2993-s001.pdf]

Supplementary data

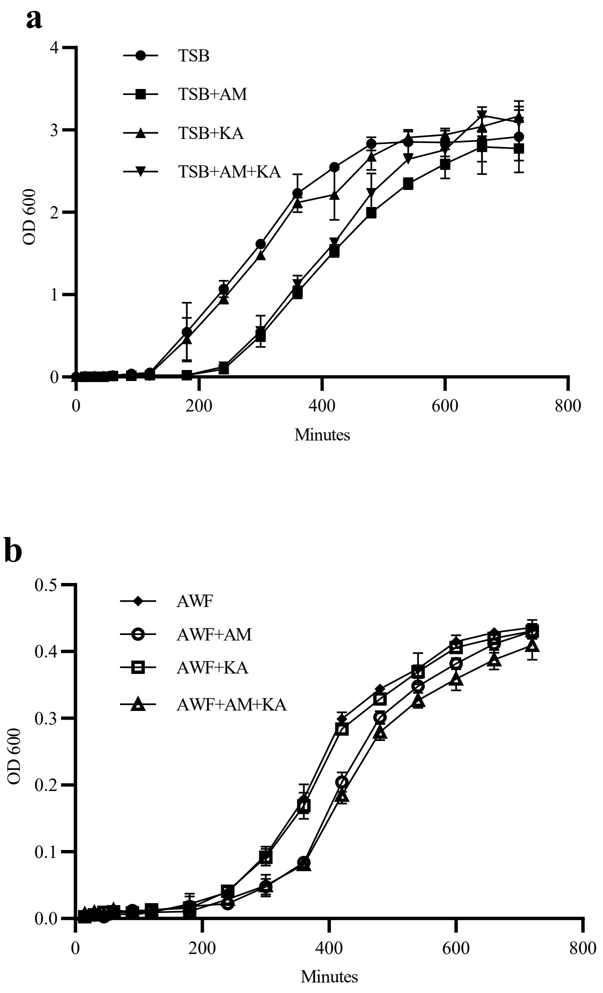

Supplementary Figure S1. Growth curves of *S. aureus* MRSA252 in TSB and AWF with or without ampicillin and kanamycin at 37 °C with shaking at 180 rpm, where a) is *S. aureus* MRSA252 growth curves in TSB and AFW; b) is growth curves in TSB with/without antibiotics; and c) is growth curves in AWF with/without antibiotics. The curves show the means of optical density at 600 nm, and error bars indicate standard deviations. Each experiment was done in duplicate. TSB, tryptone soy broth; TSB + AM, tryptone soy broth with ampicillin; TSB + KA, tryptone soy broth with kanamycin; TSB + AM + KA, tryptone soy broth with ampicillin and kanamycin; AWF, artificial wound fluid. Both a and b show the mean values from 2 independent experiments.

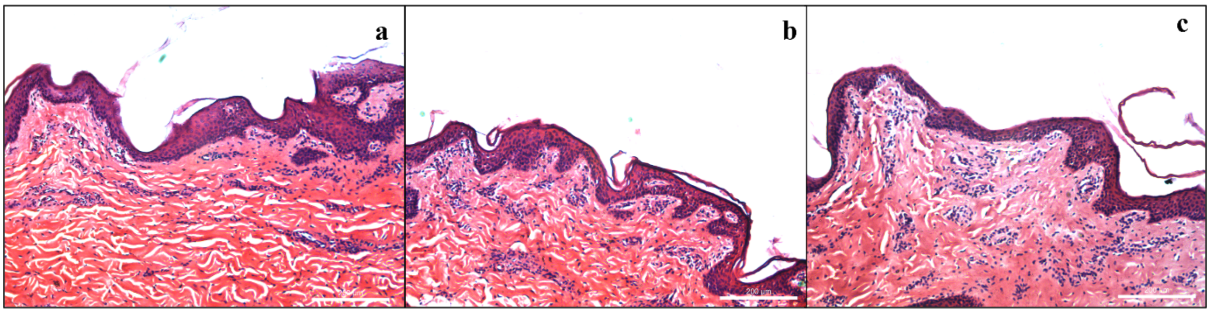

Supplementary Figure S2. Histology of untreated (a), PBS treated (b) and 0.1% PAA treated (c) porcine skin pieces. The scale bar is equivalent to 200 μm.
